# Trust and willingness towards COVID-19 vaccine uptake: a mixed-method study in Ghana, 2021

**DOI:** 10.1186/s13690-022-00827-0

**Published:** 2022-02-21

**Authors:** Joshua Amo-Adjei, Anastasiia Nurzhynska, Ruth Essuman, Anna-Leena Lohiniva

**Affiliations:** 1 Department of Population and Health, University of Cape Coast, Cape Coast, Ghana; 2 UNICEF, Ghana Country Office, Accra, Ghana; 3 Kantar Public, Ghana Country Office, Accra, Ghana

**Keywords:** Trust, Vaccine uptake, Willingness, Ghana

## Abstract

**Background:**

On the account of limited doses of COVID-19 available to the country, the Government of Ghana created a priority list of persons to target for its vaccination agenda. In this paper, we look at trust and how it informs willingness to take the COVID-19 vaccine among persons targeted for the first phase of COVID-19 vaccination program in Ghana.

**Methods:**

A sequential mixed-method investigation was conducted among the priority population - persons 60 years and above, frontline government functionaries, health workers, persons with underlying health conditions and, religious leaders and teachers. We sampled 415 respondents from the target population for a survey and 15 religious and traditional leaders from three cities; Accra, Cape Coast and Tamale for follow-up in-depth interviews based on the results of the survey data. Quantitative data is presented with descriptive proportions and multinomial logistic regression and thematic approach is applied to the interview data.

**Results:**

Trust and willingness to take the vaccine are high in this priority population. Trust in the effectiveness and safety of the vaccine, rather than socioeconomic characteristics of respondents better predicted acceptance. From interview narratives, mistrust in political actors - both local and foreign, believe in superior protection of God and seeming misunderstanding of vaccine development processes countermand acceptance. On the other hand, the professional influence of people in one’s social networks, and past triumphs of vaccination programmes against concerning childhood diseases embed trust and acceptance.

**Conclusions:**

Attention ought to be given to trust enhancing triggers while strategic communication approaches are used to remove triggers of mistrust.

## Background

The sudden outburst of the novel coronavirus in late 2019 and early 2020 unsettled the global health landscape. To end the pandemic, the pace of global vaccine response to develop effective vaccines has been impressive. More than one year into WHO declared the corona virus disease (COVID-19) as a pandemic of global concern, there are currently effective vaccines to reduce the rate of spread, and fatal outcomes (hospitalization and death) [1]. Like all other infectious diseases, widespread endorsement of the vaccine is a critical step towards decelerating the spread of the virus with positive net cascade on herd immunity [2].

While accepting vaccines is considered an individual responsibility, it is also a right, which individuals and communities need to appreciate and demand immunization services [3]. Yet, many people deliberately do not appropriate their right to vaccines. Broadly described as vaccine hesitancy, MacDonald [4] defines it as the “delay in acceptance or refusal of vaccination despite availability of vaccination services … within a specific context, varying from across time, place and vaccines which is underpinned by complacency, convenience and confidence”.

Prior research identifies trust as an important concern/attribute of vaccine hesitancy [4, 5].Trust in the context of vaccine uptake represents a relationship that exists between individuals, as well as between individuals and a system, in which one party accepts a vulnerable position, assuming the best interests and competence of the other, in exchange for a reduction in decision complexity [6]. Whereas the record time development of different vaccines are celebrated, there are widespread conspiracies around the emergence of COVID-19 and this continues to share attitudes towards and acceptance of the vaccine [7], regardless of the preponderance of evidence that affirm the potency of all vaccines currently in use [8, 9].

Immediately after different COVID-19 vaccine candidates were registered for clinical trials, a couple of studies were conducted to understand the link between hesitancy and approval of vaccines for COVID-19. For example, a recent systematic of 31 peer-reviewed published studies found an average trust/acceptance range of 23.6–97% among the adult general population and 27.7–78% among health workers. The review also noted that the scope of studies emanating from some world regions – including sub-Saharan African remained scant [10].

In sub-Sahara Africa and other lower middle-income countries (LMIC) some of the documented evidence on COVID-19 vaccine highlight personal protection against infection as a key reason for vaccine acceptance. However, concerns persist around potential side effects [11]. Specifically in Ghana, Acheampong, Akorsikumah [12] found that slightly more than half (51%) of their respondents were likely to take COVID-19 vaccine if generally made available. The remainder was undecided (28%) or flatly unlikely (21%) to accept the vaccine. The differences in acceptance were characterized by age, gender, and sources of information on COVID-19.

This paper builds on our current understanding of COVID-19 specific hesitancy. Currently, much of the evidence was generated prior to the large-scale global deployment of vaccines [13]. Also, much of the evidence that we know currently about COVID-19 vaccine trust is based on data collected prior to the approval of the vaccine candidates that were undergoing trials. The implication is that survey respondents were interviewed based on hypothetical scenarios [14]. With availability of approved vaccines, it is important to understand the extent/level of trust in actual vaccines.

On 24th February 2021, Ghana received the first consignment of vaccines for COVID-19 through the WHO COVAX platform. With a limited number of vaccines, the Ghana Health Service developed priority guidelines for the vaccination programme. The categories of the population prioritized in the first phase were: health workers, people aged 60 years and above, persons with underlying conditions, frontline executive (including security personnel), judiciary, legislature, and teachers. The first phase of nationwide deployment began on March 1^st,^ 2021 with the President Nana Akuffo Addo being the first Ghanaian to receive the vaccine. This action was partly to boost the confidence and trust and eventual acceptance by the majority of the population. As of 24th June 2021, Ghana had received 1.23 m doses of Astrazeneca vaccines with 381, 000 people fully vaccinated, representing 1.3% (compared to 10% globally) of the country’s population. As part of efforts to support continuation of successful vaccine deployment in Ghana, this rapid assessment study investigated trust in COVID-19 and how it translates into acceptance/willingness towards uptake. Specifically, two questions are asked: what factors drive trust in the COVID-19 vaccine and how does trust shape vaccine acceptance intentions.

### Conceptualizing trust and linkages with vaccine uptake

The success of vaccination uptake hinges strongly on the trust people have about the safety and efficacy of vaccines, trust in the health professionals who administer vaccines, the wider health system and the political environment within which vaccine development and deployment decisions are made [15–17]. In more general terms, Misztal [18] describes trust as believing amidst uncertainty. And because of the uncertainties of risk, some scholars point to the rationale character of trust which pushes the discourse beyond risks to questioning the trustworthiness of institutions [19]. Trust occurs between people, people and organizations and people and events as being cognitive/rational gamble (assumption that the other person/agent will act in your best interest) and affective/altruistic (emotional ties or shared values and the believe that the other will not harm you) [20].

Larson, Clarke [6] views vaccine-related trust as “relationship that exists between individuals, as well as between individuals and a system, in which one party accepts a vulnerable position, assuming the best interests and competence of the other, in exchange for a reduction in decision complexity” (p. 1599). The underlying assumption here is that there is power imbalance between the trusting party and the trustee on account of information asymmetry [6]. Decision-making under such scenarios is informed by risk-benefit analysis given that the one taking the vaccine is not endowed with complete information [21]. Following a systematic review of evidence on vaccine trust, Larson, Clarke [6] identified *trust in the vaccine*, *the provider*, and *the policy maker* (i.e., health system, government, public health professionals connected with approving vaccines) as key levers. Other dimensions include trust in the information and education on vaccines (e.g., source, channel) [6]. There other levers of trust which are external to vaccine itself. Generalized trust, historical drivers and external drivers are further described briefly.

Generalized trust is related to the extent to which people in a community are willing to trust each other [22]. Viewed as a form of social capital, it highlights how community-mindedness and civic commitment promotes generalised trust, which is underpinned by the presence of fair and efficient social institutions as well as the efficiencies in deterring acts that are communally agreed to be wrong. In short, generalised trust is attained and internalised at the communal level due to efficiencies in social institutions and their agents [23]. Relative to vaccination programs, this is more concerned with information sharing from official institutions to members of the society. If the information is believed to be accurate (dependent on many considerations such as the credibility of the institution and its people), generalised trust will advance vaccination acceptance and uptake.

Another external lever is historical influences on trust. The perceptions about the past performance and the values of a health system are believed to uphold historical influence [6]. In relation to vaccination programs, historical influences are driven by social trust (defined as shared values of benevolence, fidelity and morality) and confidence (performance-based; belief in the confidence and capability of the trusted individual). In public health, populations that have historically experienced or perceived to be victims of medical injustices and everyday discriminations may view vaccines as another mischievous/negative agenda against their communities [24]. Within the medical trust/mistrust literature, the Tuskegee [25], Sims [26] and Lacks [27] studies are frequently cited, even though some evidence (e.g., [28, 29]) downplays its role in racial disparities in vaccine uptake in US, for instance.

The last strand/lever is external influences which are primarily linked to the sources of information predicating decision-making on vaccination. According to Larson, Clarke [6] this borders on the motive of the source of information – whether it is considered altruistic and the second is ability – the perception that the source has been competent in the past on related matters. These influencers include friends, family members, non-official medical advice such as from religious networks, alternative health networks, politicians and celebrities.

## Data and methods

We utilized a sequential mixed method design to generate data for this paper. Data from a quantitative survey are presented, and complemented by qualitative data. The quantitative survey focused on the first categories of people profiled to be vaccinated in the first phase of the vaccination programme. These targets were health workers, teachers, persons aged 60 years and above, frontline security personnel, people with underlying health conditions, national level religious leaders, and frontline executive, judiciary and legislature (e.g., ministers of state). A non-probability sampling approach was utilized, given the near-impossibility of following probability techniques during pandemics [14]. We adopted a quota sampling approach mainly to align with the Ghana Health Service targeting for the deployment of the vaccine. For health workers, we obtained a list from Ghana Health Service based on which random samples were drawn. For the rest of the target population, snowballing was applied. Specifically, used exponential non-discriminative technique which allowed initial contacts to provide multiple referrals. We estimated a sample size of 384 based on the assumption that a minimum of 50% of the targeted population will accept the vaccine based on Dean, Sullivan [30]. The 50% is a conventional marker applied in finite population when the prevalence of an outcome is unknown for a simple random sampling process. A 10% of the estimated sample was added to make room for non-response. The analysis is based on 415 usable respondent data. A structured questionnaire was uploaded onto tablets (computer-assisted personal interviewing – CAPI) and administered to respondents. Questionnaire administration lasted an average of 20 min on telephone. Response rate for health workers was 85% while those targeted through snowballing yielded a response rate of 72%. As the recruitment was through telephone, the main reason for refusal was often about the calling time conflicting with other schedules of the study population. Enumerators and supervisors were trained for three days on the context, content of the tool, and protocols for conducting successful interviews, including ethics and informed consent.

The survey data was analyzed using descriptive and inferential statistics. The descriptive statistics – proportion and Chi-square were used to determine associations between explanatory and outcome variables. Next, we used a multinomial logit model given that the two outcome variables – willingness to accept COVID-19 vaccine is polytomous in nature (yes = 1, no = 2 & not sure = 3). The multinomial allows us to approximate the probability of an event occurring using the maximum likelihood function. The multinomial model generates a K-1 set of parameter estimates and compares different categories/outcomes on the dependent variable to a certain base category/outcome [31]. We used “not sure” response as the base category for this analysis as the outcome with the least frequency of responses.

From the quantitative survey, we found that a comparatively high proportion of religious leaders were not willing to take up the vaccine. It is within this context that religious leaders were chosen for further exploration using qualitative methods. Focusing on this subset of the larger sample is also justified because of the religious dimension of the country’s initial response to the pandemic. That Ghana is “deeply religious” which pervades all national life is well documented [32]. Further religious interpretations are placed on uncommon events including sudden epidemics [33–35]. Not surprisingly, the President declared a national day of fasting for God’s intervention and peradventure, and to forgive the sins of the nation [36]. The second, and equally important is the substantial influence religious leaders tend to have on their followers in Ghana [37]. Subsequently, when the government started preparations to receive the first batch of COVID vaccines, religious leaders were called on to encourage their congregants to accept and take up the vaccine. We interviewed 15 religious (Christian and Muslim) and traditional leaders in three areas: Accra, Tamale and Cape Coast. We ensured that the Christian leaders, in particular, reflected diverse backgrounds; mainstream Orthodox Christian groups (e.g., Methodist, Pentecostal, Catholic), Islam, and African Syncretic churches. Interviews were conducted in either English or Twi, Ga or Fante. Three experienced qualitative moderators conducted interviews within a period of four weeks. Interviews were conducted within participants’ home and office environments where confidentiality and privacy of conversations could be maintained. The duration of interviews ranged from 30 to 45 min. The IDI tool explored the following themes: information and communication on COVID-19, perceptions about COVID-19, and trust of COVID-19. The interview recordings were transcribed verbatim and edited for basic grammatical errors before being analyzed. The edits were undertaken by the lead data analyst with validation by the interviewers. The qualitative component was conducted in April and May 2021.

The analysis of qualitative followed the framework approach (familiarization, identifying a thematic framework, indexing, charting and mapping and interpretation) to qualitative data analysis proposed by [38]. Three experienced qualitative researchers coded the data independently and all authors reviewed the draft report vis-à-vis the transcripts for consistency and consensus. Specifically, each of these coders read all the transcripts as part of the familiarization process. The second stage involved identifying recurrent issues and themes. In the next phase, the themes were refined (indexing) and proceeded to summarize into concise and coherent forms. The final stage was used to compare themes and subthemes respondent categories with transcripts, field notes and tape recordings where necessary. NVivo 12.0 was used to facilitate the coding processes.

## Results

Of the 415 respondents surveyed, approximately 53% identified as males. Around two-thirds of respondents were below 40 years; the majority (73%) of respondents reported higher or tertiary level education and about 76% indicated employment in the formal sector. Around half (51%) of respondents expressed moderate trust in the vaccine and about 34% indicated they very much trust in the vaccine; the rest (14%) had no trust in COVID-19 vaccine. A higher proportion of respondents (70%) stated willingness to take the vaccine if made available to them; 20% will not accept and 10% were undecided.

We proceed further by looking at the specific background characteristics of respondents and the level of trust in the vaccine, with the corresponding Chi-square values. Age shows a significant association with level of trust, with the highest proportion of trust observed among respondents 50–59 years (~ 58%) and those 60 years and above (57%). The results do not show significant association between males and females; only about one-third each of males and females expressed high levels of trust in the COVID-vaccine. The association between educational attainment and trust is moderately significant (χ^2^ = 12.90; *p <* 0.045). Also, the sector of employment has significant association with levels of trust with a comparatively higher proportion of those working in the informal sector (28%) reposing no trust relative to the unemployed/students (12%) and those in the formal sectors (11%). Among the categories of people targeted for vaccination in the first phase, the highest share of respondents who have no trust in the vaccine were religious leaders (42%). On the other hand, health workers (45%) were more inclined to express higher levels of trust (45%). Other results on this item are available in Table 1.

**Table 1:** Background and trust in COVID-19 vaccine among first phase COVID-19 vaccination target population in Ghana, 2021

	Extent of trust in COVID-19 vaccine			
**Respondent characteristics**	No trust	Moderate trust	Very much	Total
**Age** (χ^2^ = 41.24; *p <* 0.000)				
< 20–29	20.2	62.8	17.1	129
30–39	13	50.7	36.2	138
30–34	16.4	52.7	30.9	55
50–59	2.5	40	57.5	40
60+	11.3	32.1	56.6	53
**Sex** (χ^2^ = 2.37; *p <* 0.304)				
Female	11.7	52	36.2	196
Male	16.9	50.7	32.4	219
**Level of education** (χ^2^ = 12.90; *p <* 0.045)				
No formal edu	14.3	28.6	57.1	7
Basic Education	30	26.7	43.3	30
Sec/Voc	16.4	53.4	30.1	73
Higher/Tertiary	12.5	53.8	33.8	305
**Sector of employment** (χ^2^ = 14.75; *p <* 0.005)				
Informal Worker	28.4	40.5	31.1	74
Formal Worker	11.4	54.1	34.5	316
Unemployed/Student	12	48	40	25
**Vaccine deployment group of respondents** (χ^2^ = 34.47; *p <* 0.001)				
60 years & above	20	70	10	20
Essential worker	20.3	46.9	32.8	177
Frontline executive/security personnel	9.4	71.9	18.8	32
Health workers	4.2	50.4	45.4	119
People with underlying conditons	12.5	37.5	50	8
Religious leader	42.9	42.9	14.3	7
Teacher	15.4	51.9	32.7	52
**Total**	**14.5**	**51.3**	**34.2**	**415**

The next item we assess is willingness to take up COVID-19 vaccine. A statistically significant association is noted between level of trust and willingness to accept the vaccine; 69% and 98% of respondents who moderately and very much trusted the vaccine were willing to take up. The contrary is the case for those who do not trust the vaccine; 73% of this group will not accept the vaccine. Vaccine deployment group (χ^2^ = 44.62; *p <* 0.000), age of respondents (χ^2^ = 27.77; *p <* 0.001) and sector of employment (χ^2^ = 16.25; *p <* 0.003) [Table 2] showed significant association with vaccine acceptance. It is particularly instructive to note that almost two-thirds (57%) of religious leaders sampled would not accept the vaccine; the highest in any category in the prioritized populations.

**Table 2:** Willingness to take COVID-19 vaccine among first phase COVID-19 vaccination target population in Ghana, 2021

	Willingness to take COVID-19 vaccine				
	No	Yes	Not sure	Total	Total
**Level of trust in COVID-19 vaccine** (χ^2^ = 175.93; *p <* 0.000)					
No trust	73.3	10	16.7	100	60
Moderate trust	17.4	68.5	14.1	100	213
Very much	1.4	97.9	0.7	100	142
Total	20	70.1	9.9	100	415
**Vaccine deployment group of respondents** (χ^2^ = 44.62; *p <* 0.000)					
60 years & above	35	45	20	100	20
Essential worker	26	60.5	13.6	100	177
Frontline executive/security personnel	18.8	75	6.2	100	32
Health workers	5.9	88.2	5.9	100	119
People with underlying conditons	12.5	62.5	25	100	8
Religious leader	57.1	42.9	0	100	7
Teacher	23.1	73.1	3.8	100	52
**Age of respondent** (χ^2^ = 27.77; *p <* 0.001)					
< 20–29	31.8	57.4	10.9	100	129
2. 30–39	21	68.1	10.9	100	138
3. 30–34	12.7	78.2	9.1	100	55
4. 50–59	2.5	87.5	10	100	40
5. 60+	9.4	84.9	5.7	100	53
**Sex** (χ^2^ = 4.1; *p <* 0.128)					
Female	15.8	73.5	10.7	100	196
Male	23.7	67.1	9.1	100	219
**Level of education** (χ^2^ = 9.37; *p <* 0.154)					
No formal edu	14.3	71.4	14.3	100	7
Basic Education	33.3	56.7	10	100	30
Sec/Voc	28.8	61.6	9.6	100	73
3. Higher/Tertiary	16.7	73.4	9.8	100	305
**Sector of employment** (χ^2^ = 16.25; *p <* 0.003)					
Informal Worker	33.8	52.7	13.5	100	74
Formal Worker	16.1	75	8.9	100	316
Unemployed/Student	28	60	12	100	25
Total	20	70.1	9.9	100	415

In the qualitative study, five participants were Christian leaders, 3 Muslim leaders and 6 traditional leaders, a total of 14 participants. From the qualitative data, we note both positive and negative accounts of trust in the vaccine (COVID-19) and how they connect with intentions to accept the vaccine or otherwise. On the positive note, some participants drew on past successes of public health vaccination programmes to ground their confidence in the COVID-19 vaccine. Those who shared this view noted that vaccines were not new to the global health landscape. Participants of this view recounted “nostalgic” memories of the scourge of some childhood diseases and their communities faced constant threats of diseases such as polio and measles. To these participants, mass vaccination programmes have helped to almost eradicate these diseases. They did not see the COVID-19 vaccine as any different from vaccines in use now except that this (COVID) is new and perhaps the cause of people’s apprehension. A traditional religious leader elaborated:

*They are vaccines (COVID-19) that make life better…Polio vaccines are still in the system and we encourage all to get vaccinated. The first polio vaccine was done with a knife, it was so painful, it caused us stress (headache), then another one came which looked like a gun and was shot into the arm. Then came the needle, that one was calm and gentle, but they could inject about 20 people with one needle, later they said one person to a needle. We encourage people to go for those vaccines, so I support vaccination of any kind (Mosque Leader, Tamale).*

We also heard accounts of lack of trust in COVID-19 vaccines. Participants gave several key propositions to support views. One of these was low pandemic risk perception. Some participants did not consider COVID-19 a major health concern for Ghana given that the country has recorded relatively low deaths associated with the pandemic. To them, it did not make sense to by-pass those most affected by the pandemic in other countries (in terms of caseload and fatality rates) to supply vaccines to Ghana. This made them skeptical about the intentions of vaccine manufacturers as the government, feeding into the conspiracies around COVID-19 and its vaccines. These participants argued that the “West” where the vaccines originate are not genuinely interested in Africa and extension, Ghana. One church leader in Cape Coast questioned:

*I don’t trust it at all. Because those who are being killed by the virus are abroad so why don’t they go and give it to them? They have different mind-set to kill Ghanaians. (Charismatic Church Leader, Cape Coast)*

Other accounts asserted mistrust due to the origins of the vaccines and expressed preference for locally produced vaccines as illustrated in the excerpt below:

*For me, the source of the vaccine is what is of concern. This is because already they are not after our interest. I don’t trust those foreign countries. Besides, if they produce the vaccine here, our people can monitor and ensure its safety right from the production process. Look, where these vaccines are coming from, they are not after our welfare. They are wicked people. I don’t trust them! The same people who brought this COVID-19 disease are the people who are now bringing these vaccines. So I asked myself, why did they bring this disease to the world in the first place? We have a lot of diseases that occur naturally. But, for this COVID-19, we were made to understand that it was a virus from the lab in China. So, it is difficult for me to accept a lot of things like the vaccine and things like that (Pentecostal Church Leader, Cape Coast).*

The second source of mistrust in COVID-19 vaccine arose from lack of trust in the government and political leadership that is leading the fight against the pandemic. Participants who aligned with this proposition contended that politicians presented different narratives on issues depending on the season. This general sense of mistrust in political leadership made one participant for instance, to question whether the live vaccination of the President was real. This participant showed some conviction that the President must have been given a placebo to shield him from any side effects of the true vaccine. He surmised:

*When the vaccine was brought initially, it was the President who went to receive the first dose. But I have my doubts as to whether what he received was the vaccine or something else. Politicians don’t tell the truth. There is no truth in Ghana. It could be that it wasn’t the COVID-19 that was given to the President, but rather a different injection altogether; it could even be just water [laugh]. They are only staging it to encourage people to go and receive the vaccine. So, for the vaccine, it will be very difficult; it is very difficult actually. (Charismatic Church Leader, Cape Coast)*

Another concern noticed in the data was how lack of knowledge on vaccine development, which has heightened mistrust. Some participants narrated that they had heard stories which suggested that the vaccines were developed using weakened forms of the corona virus. In the views of these participants, they are discouraged from taking the vaccine because they feared that the introduction of the virus in its weakened state could trigger more fatal diseases or hidden conditions. Worse still, the view that vaccines do not provide absolute protection and that vaccinated people could still get infected meant there was no need to get vaccinated. On account of this, some are hesitant:

*I’ve heard from WhatsApp that the vaccine is made from the virus that causes the disease. So, if you take the vaccine, it will affect you. So, we shouldn’t take it. Later, one of those celebrities explained that when it comes to the vaccine, assuming the virus is 100%, they will take about 10% of the virus to produce the vaccine so that if it is given to someone, the person’s body will fight it and get used to it. That means it has defeated the virus so anytime a new one enters the body, the body will fight it. When I heard that, I said to myself that I don’t have the disease; I am not infected with the disease, why then should I go and allow that 10% into my body? That wouldn’t be a wise decision. After all, we were told that even after receiving the vaccine, you still have to continue using the nose masks and the hand sanitisers and practising social distancing. So, even with or without the vaccine, we still have to go ahead and use preventive measures (Religious Leader, Cape Coast)*

From the descriptive analysis, we constructed two multinomial logit models with one having only trust and a second where we adjusted for age, sex, level of education, sector of employment and vaccine deployment category. The results are presented in Table 3. Models 1 (No) and 3 (Yes) are bivariate exploring the linkages between trust and vaccine acceptance. Regression results generally align with the descriptive findings. For respondents who are not willing to accept the vaccine (Model 1), the level of trust (no trust, moderate and very much) did not significantly change the direction of the relationship – all levels of trust were negatively associated with vaccine acceptance. Controlling for other factors in Model 3, the coefficients remain unchanged (Table 3). On the reverse, willingness to accept the vaccine is strongly predicated on trust, and the results are consistent across the bivariate (Models 2 & 4). Substantially significant positive coefficients are recorded among respondents with moderate trust (Coef.=2.93; CI = 1.01–3.18) and full trust (Coef.=5.44; CI = 3.23–5.67) in the vaccine. adjusting for the control factors, direction and signs remain unchanged (Model 4).

**Table 3:** Multinomial logistic regression on trust in COVID-19 vaccine and willingness towards uptake among first phase COVID-19 vaccination target population in Ghana, 2021

	*Willingness to take vaccine (No)*				*Willingness to take vaccine (Yes)*			
	*Model 1*		*Model 2*		*Model 3*		*Model 4*	
	Coef.	*95% CI*	Coef.	*95% CI*	*Coef.*	*95% CI*	*Coef.*	*95% CI*
***Trust (No trust)***	*0*	*[0,0]*	*0*	*[0,0]*	*0*	*[0,0]*	*0*	*[0,0]*
* Moderate*	*-1.272* ^**^	*[-2.111, -0.433]*	*-1.259* ^**^	*[-2.194, -0.323]*	2.093^***^	*[1.008,3.179]*	2.161^***^	*[0.983,3.339]*
* Full trust*	*-0.788*	*[-3.285,1.708]*	*-0.397*	*[-3.026,2.232]*	5.445^***^	*[3.233,7.657]*	5.669^***^	*[3.291,8.047]*
	1.482^***^	*[0.795,2.168]*			*-0.511*	*[-1.523,0.501]*		
***Vaccine deployment group of respondents*** *(60 years and above)*								
* Essential worker*			*1.259*	*[-1.108,3.626]*			*-0.0356*	*[-2.215,2.144]*
* Frontline executive/Security personnel*			*1.739*	*[-1.141,4.620]*			*1.379*	*[-1.241,4.000]*
* Health worker*			*1.042*	*[-1.563,3.647]*			*1.260*	*[-1.029,3.549]*
* People with underlying conditions*			*-0.215*	*[-3.530,3.100]*			*-1.046*	*[-3.904,1.813]*
* Religious leader*			*14.42*	*[-1022.2,1051.0]*			*12.17*	*[-1024.4,1048.8]*
* Teacher*			*2.326*	*[-0.470,5.123]*			*1.651*	*[-0.937,4.238]*
***Age*** *(< 20–29)*			*0*	*[0,0]*			*0*	*[0,0]*
* 30–39*			*-0.453*	*[-1.465,0.559]*			*-0.285*	*[-1.219,0.649]*
* 40–49*			*-0.941*	*[-2.416,0.534]*			*0.625*	*[-0.669,1.919]*
* 50–59*			*-2.231*	*[-4.613,0.152]*			*0.0539*	*[-1.365,1.473]*
* 60+*			*-0.969*	*[-2.819,0.882]*			*1.424*	*[-0.244,3.092]*
***Sex*** *(Female)*			*0*	*[0,0]*			*0*	*[0,0]*
* Male*			*0.424*	*[-0.430,1.279]*			*0.247*	*[-0.542,1.036]*
***Level of education*** *(No formal education)*			*0*	*[0,0]*			*0*	*[0,0]*
* Basic education*			*1.199*	*[-2.145,4.542]*			*1.279*	*[-1.748,4.305]*
* Sec/Voc*			*0.944*	*[-2.260,4.147]*			*1.026*	*[-1.716,3.768]*
* Higher/Tertiary*			*0.341*	*[-2.815,3.497]*			*1.224*	*[-1.470,3.918]*
***Sector of employment*** *(Informal worker)*			*0*	*[0,0]*			*0*	*[0,0]*
* Formal worker*			*-0.147*	*[-1.405,1.110]*			*-0.0137*	*[-1.211,1.184]*
* Unemployed/Student*			*1.055*	*[-1.622,3.732]*			*-0.149*	*[-2.600,2.303]*
* _cons*			*-0.123*	*[-4.055,3.810]*			*-2.541*	*[-6.052,0.970]*
* Log lik.*	*-240.5*						*-213.1*	
* Chi-squared*	*182.5*						*237.3*	
* N*	*415*						*415*	

Base category – Not sure; 95% confidence intervals in brackets; ^*^ *p* < 0.05, ^**^ *p* < 0.01, ^***^ *p* < 0.001

Data from our qualitative interviews revealed that some of the participants (about three) had been vaccinated at the time of the interviews. Others intended to take up the vaccine when it was their turn; three participants had refused to take the vaccine. We note that participants’ source of information about the vaccine contributed to the decision to accept the vaccine. This is often the case when the source of information was personally connected to the target of information. These connections varied from participant to another. However, religion, ethnicity and political relationships appeared pronounced from the data. For instance, one religious leader together with his wife took the vaccine. Their decision was triggered and motivated by a church member who is also a health professional. Apart from the education he had received on the public health benefits of the vaccine, another critical element in the decision-making process was the comfort and assurance that his church was not inclined to recommend something that could harm or damage his health. He briefed:

*I took it because of the recommendation. I trust the one who recommended it to me. As I said earlier, she is a health worker and a member of the Church so I trust her. Moreover, based on the things that I have read, I realized the things she was saying were true as they were consistent with the things I’ve read. So, I will say that I took the vaccine based on the fact that it was recommended to me by someone that I trust. (Methodist Church Leader, Cape Coast)*

For those yet to take the vaccine, the motivation was due to the fact that no one who has taken up the vaccine in the country had reported any fatal outcomes days after the vaccine roll-out. To them, this has fastened their trust and confidence in accepting the vaccine. A female religious leader in Accra opined:

*I don’t have any problem with taking the vaccine because as far as I know, many people have been taking the vaccine and nothing has happened to them. There is an adage that says prevention is better than cure; it is better to prevent it than to have it and go for a cure. So personally, I think there is nothing wrong with it; to prevent it before it is too late. (Church Leader, Mamprobi, Accra)*

It is also worth noting that while some religious leaders did not discount their own and households’ vulnerabilities to COVID-19, there was constant reference to the absolute protection that God offered. This sense of protection, in their views, surpassed the effectiveness of vaccines and if the vaccine was offered to them, they would first consult (pray for directions) God before taking it up. When asked whether their religious practices forbade vaccination, all answered in the negative and further affirmed the rights of individual church members to make their own decisions on vaccine uptake or otherwise.

## Discussion

It now looks reasonable to expect that COVID-19 will remain on the global public health landscape for some time. Fortunately, the evolving evidence so far points to sustained benefits of vaccines, particularly against severe cases, hospitalization and mortality [39–42]. While preventive behaviours such as wearing of face masks, frequent washing, stay home orders, avoiding crowded places among others are useful mechanisms to slow down the pace and reach of infections, the sustainability of these measures are interrogated on the grounds of political and economic survival, food security, and challenges of consistency in human behaviours [43–45]. Large-scale and widespread vaccination against the pandemic offers a more sustainable and long-term relief from the disease than other measures such as lockdown. Vaccination is one of the most effective primary preventive interventions in public health. We studied some of the possible undergirding around trust/mistrust in COVID-19 among the category of people targeted in Ghana’s first phase vaccine deployment. Survey data showed moderate (51%) to high (34%) trust among the respondents and 70% intended to get vaccinated – similar to levels reported in Australia [46]. From the qualitative study, we find varied undertones of trust and mistrust in the vaccine. Trust in this study is historical (past triumphs in vaccination programmes), influenced by trusted social networks – of those personally connected and information from perceived altruistic public figures. Mistrust on the other hand was shaped by lack of confidence in political authorities, beliefs in negative consequences of vaccines, concerns about efficacy, conspiracy theories and low understanding of vaccine development processes.

Overall, our quantitative results present a positive scenario around COVID-19 vaccine trust and willingness to take the jab. Willingness to accept was substantially pushed by trust in the vaccine. Of the sociodemographic variables investigated, none was significantly related to willingness to take COVID-19 vaccine contrary to observations in the descriptive section. These findings are contrary to some of the emerging studies on willingness to accept COVID-19 vaccine. Particularly striking is the absence of gender differences (in favour of men reporting higher acceptance indicators) which rather seems a consistent finding across several studies [47]. Evidence thus far points to higher fatal outcomes (hospitalization and mortality) among men compared to women [48, 49]. The higher likelihood of men intending to vaccinate maybe related to their higher risk of COVID-19 infection and the corresponding poorer outcomes. However, the comparatively low levels of reported cases and deaths in the country, the lack of gender differences may be understood in that light.

From our qualitative data, it becomes evident that trust and willingness to accept the vaccine is nuanced. For instance, among those who express trust in the vaccine, past triumphs of vaccination programmes against endemic and concerning childhood diseases was an important lever in their intentions to accept COVID-19 vaccine. Such accounts reveal some deep sense of “celebration” of the vaccine successes. This was evident even among some participants who expressed hesitancy. These positive memories associated with vaccination interventions for other diseases considered troubling could be harnessed for behavioural change communication around COVID-19 vaccination. This observation aligns with an earlier proposition by Stern and Markel [50], stating that anti-vaccination narratives are not always towards all vaccines but specific vaccines. By invoking such memorable previous feats over diseases people can easily relate to, campaigns towards higher acceptance rates may be achieved.

Our findings also illustrate and highlight the capital and opportunities to deploy early adopters [51] as champions to share their success stories about their experiences with the vaccine. From the qualitative data, some participants expressed willingness to take the vaccine given that there is no reported incidence of any unusual or extreme side effect among the first batch of Ghanaians who had taken the vaccine. This can help allay the fears associated with the vaccine. However, this can become tricky if some of the early adopters are people who are mistrusted by a section of the population. The qualitative findings specifically highlight the lack of trust some show towards the political class, some of whom were in the early adopters’ category. The alternative is to utilize the power of personalized communication of health professionals with targets with whom some social, cultural, religious etc. connections exist. As shown in this report, certain participants had taken up the vaccine due to prodding of people they connected closely on religious grounds. This finding aligns with the earlier seminal work of Larson, Jarrett [15] on the broad determinants of vaccine hesitancy or acceptance which includes the influence of social, cultural and political institutions.

The findings also point to how misunderstanding of information around vaccine development may negatively affect vaccine uptake intentions. Similarly, mistrust in institutions – political and medical that produce vaccines also shape intentions [52]. Briefly, these discourses, described as *bad pharma* [53, 54] entail concerns about expenses of pharmaceutical firms where more allocation is made to marketing than research and development [55], profit upturn [56, 57] and disease mongering or selling sickness [58, 59], accusations of manipulation of trial protocols and reporting of negative results and lack of transparency in pricing regulations, collaborations with researchers and academic institutions [60–62]. Coupled with some ‘misunderstandings’ of vaccine development processes, the qualitative evidence also highlights concerns around the motive of pharmaceuticals or more broadly, political institutions in pursuing COVID-19 vaccination against a perception that countries such as Ghana do not have COVID-19 crisis compared to other Western nations and therefore unjustifiable to promote vaccination. For some participants, this was more concerning given that other COVID-19 measures such as wearing of nose masks and frequent handwashing could not be abandoned even after vaccination. Rather than viewing such COVID-19 anti-vaccinationist as irrational and unscientific, it is important to recognize their fears and anxieties and instead deploy persuasive communication strategies to gain their confidence [50]. An approach to doing this could be highlighting more strongly the effectiveness of the vaccine in minimizing fatal outcomes such as hospitalization and the risk of death rather than preventing infections entirely as some viewed.

Despite the important findings we highlight in this paper, we acknowledge several limitations too. First, by using snowballing to sample a portion of the study population, we were prone to selection bias, clustering towards some particular profile (e.g., popular and populations within convenient reach). For instance, the initial recruits were more likely to propose individuals they have cordial relationships and within their immediate social networks, including sociocultural networks. Also, we relied on a very restricted sample for the qualitative interviews. This reduces participant diversity. Nonetheless, religious leaders in Ghana preside over a rich mix of congregants whose ethnic, cultural and socioeconomic backgrounds are substantially diverse and whose perspectives shape and influence their members.

## Conclusions

Our findings generally point to a certain level of positivity around COVID-19 vaccine uptake, even though pockets of hesitancy are observed. This finding provides a positive platform for pursuing the vaccination programme through collaborations and partnerships with religious organizations in promoting vaccine uptake. The fact that health workers are considered trusted sources of information means that deliberately involving health workers in communicating the uptake of COVID-19 vaccine will be important for the campaigns.

## Data Availability

Data is currently not publicly available. However, researchers may be granted access to the data for further analysis.

## Abbreviations

WHO: World Health Organization
COVID-19: Corona virus disease
LMIC: Lower-middle-income countries
